# Strengthening Global Public Health Surveillance through Data and Benefit Sharing

**DOI:** 10.3201/eid2407.151830

**Published:** 2018-07

**Authors:** Michael Edelstein, Lisa M. Lee, Asha Herten-Crabb, David L. Heymann, David R. Harper

**Affiliations:** Chatham House, London, UK (M. Edelstein, A. Herten-Crabb, D.L. Heymann, D.R. Harper);; Walter Reed Army Institute of Research, Silver Springs, Maryland, USA (L.M. Lee)

**Keywords:** public health surveillance, global health, data sharing, benefit sharing

## Abstract

Equitable sharing of public health surveillance data can help prevent or mitigate the effect of infectious diseases. Equitable data sharing includes working toward more equitable sharing of the public health benefits that data sharing brings and requires the engagement of those providing the data, those interpreting and using the data generated by others, those facilitating the data-sharing process, and those deriving and contributing to the benefit. An expert consultation conducted by Chatham House outlined 7 principles to encourage the process of equitable data sharing: 1) building trust; 2) articulating the value; 3) planning for data sharing; 4) achieving quality data; 5) understanding the legal context; 6) creating data-sharing agreements; and 7) monitoring and evaluation. Sharing of public health surveillance data is best done taking into account these principles, which will help to ensure data are shared optimally and ethically, while fulfilling stakeholder expectations and facilitating equitable distribution of benefits.

Global outbreaks, including those of severe acute respiratory syndrome (SARS), Middle East respiratory syndrome, and Ebola virus disease, remind us that a public health event in a single location can rapidly become a global crisis. Control of infectious diseases can therefore be considered a global public good, and public health surveillance is a tool that helps achieve it. Timely sharing of public health surveillance data enables better preparedness and response, locally and globally.

## Public Health Surveillance

Public health surveillance has been defined as “the ongoing, systematic collection, analysis and interpretation of health-related data with the a priori purpose of preventing or controlling disease or injury and identifying unusual events of public health importance, followed by the dissemination and use of such information for public health action” (*1*). Public health surveillance data are often collected without requiring individual patient consent. This practice is ethically and legally justified as a part of a government’s responsibility to protect the public’s health (*2*) and as a basic interest of persons in a pluralistic society (*3*). These justifications are tempered by the state’s responsibility to use data for public health purposes only, engage stakeholders, and ensure protection of personal information.

Healthcare professionals are traditionally mandated to notify public health authorities about cases of specified diseases within a certain timeframe. The authorities then analyze the data and take appropriate action. Surveillance systems therefore tend to be the responsibility of the government. Most countries provide routine surveillance data to multilateral agencies (*4*), which analyze and disseminate information on disease trends at the regional or global level. These agencies also receive data from countries when the impact of a public health event crosses national borders, a standard of practice codified by the 2005 International Health Regulations (IHR 2005) (*5*), the international legal instrument aimed at assisting the global community to prevent and respond to public health threats that have the potential to affect populations worldwide.

## Sources of Data

Public health authorities increasingly complement notifications with laboratory data (*6*), although in practice, this practice is often limited to high-income countries because it requires considerable laboratory capacity and advanced information technology infrastructure. Syndromic surveillance, in which health-related data such as the number of consultations for a specific diagnosis are reported, is used in high- and low-income settings. In some low-income settings, nongovernment actors, such as nongovernmental organizations (NGOs), academic institutions, private companies, or foreign medical teams, sometimes fill surveillance gaps (*7*), in particular during public health emergencies in which temporary, early warning surveillance systems based on syndromic surveillance are deployed in response to an increased outbreak risk (*8*).

Increasingly, online data not necessarily collected with an a priori health objective are used for public health purposes. Online technologies that provide data for disease or event detection are known as digital disease detection (DDD) systems. Data sources include search engine and social media queries (*9*,*10*), crowd-sourced event-based information queries (*11*), machine learning (*12*), natural language processing, and geolocalization (*13*). In 2014, DDD identified the resurgence of poliomyelitis and the Ebola outbreak in West Africa before the World Health Organization (WHO) officially reported them (*14*), 2 events WHO later declared as Public Health Events of International Concern. As DDD data quality and accuracy improve, DDD will likely assume a more prominent role, particularly in settings where the infrastructure to support public health surveillance systems is lacking (*10*). However, DDD needs more systematic integration into the formal, government-owned surveillance landscape as well as ties to response mechanisms to maximize its potential (*10*,*15*). The systematic use of nongovernment, informal surveillance systems is beginning to gain traction with surveillance systems such as the WHO’s new Epidemic Intelligence from Open Sources, an event-based system receiving alerts from a range of informal sources, planned for launch in the near future (*16*). DDD data raise new ethical and legal challenges that need to be addressed as they become integrated into conventional surveillance systems (*17*). These data also create competition for resources or the generation of data that are not consistent with data from conventional surveillance systems; these factors can give rise to trust and acceptability issues. Nevertheless, evidence for the added value of these data is building, and they are increasingly being incorporated into conventional surveillance systems.

## Why Share Public Health Surveillance Data?

Public health surveillance data require timely sharing to ensure more coordinated and effective risk management for public health response (*18*). Sharing public health surveillance data between countries improves capacity for disease detection and response (*19*) and can help identify an outbreak source when national-level data cannot (*20*). This sharing can also reduce the potential or actual impact of a global health crisis. For example, the Global Influenza Surveillance and Response System, a laboratory network that shares information to detect the emergence of novel influenza viruses with pandemic potential (*21*), helped prevent SARS from becoming endemic after the 2003 outbreak (*22*). This network also improved the timeliness of the response to the 2009 influenza A(H1N1) pandemic (*21*). Besides being useful for outbreak management, sharing of routine public health surveillance data enables national and international collaboration, capacity strengthening, insight into public health system performance, and ultimately better control of infectious diseases (*18*).

Real or perceived risks, in particular those risks linked to travel and trade restrictions, can lead to a reluctance by governments to share data, leading to adverse public health and economic consequences. The 2003 SARS outbreak cost an estimated US $40–$80 billion to the global economy, with travel and tourism industries badly affected (*23*). China’s delay in sharing information about the 2003 SARS outbreak contributed to the disease’s spread and the delayed global response (*24*) as well as economic and reputation damage to China (*25*). With Middle East respiratory syndrome, the incomplete assessment of the disease origin and source has largely been attributed to a reluctance to share public health surveillance data in a timely fashion (*26*). Sharing of data helps achieve appropriate public health action while limiting risks to travel and trade. IHR 2005 is designed to ensure maximum public health benefit while keeping restrictions to a minimum (*5*). Nevertheless, public health surveillance data are not always freely shared because of perceived or real technical, political, economic, motivational, ethical, and legal barriers (*27*). Sharing public health surveillance data must become the norm.

## Which Stakeholders Are Concerned?

Government actors implement most conventional public health surveillance systems and generate most data and can be complemented by nongovernment actors. In addition to having value at the national level, a country’s routine public health surveillance data enable multilateral organizations to generate intelligence on specific diseases at the regional and global level. These organizations provide standards and advice on data sharing to facilitate the process by individual countries and conduct their own surveillance activities (*28*); examples include the WHO’s global measles surveillance system (*4*) and the European Union’s surveillance system (TESSy), which has standardized surveillance across the European Union (*29*). Such supranational systems come with their own challenges, such as the additional burden placed on individual countries to report data already analyzed nationally and the difficulties associated with comparing different types of data resulting from surveillance systems with different national legal bases. Institutions that do not generate data themselves but seek to reuse data for academic or public health purposes are also part of this data-sharing landscape (Figure).

**Figure:**
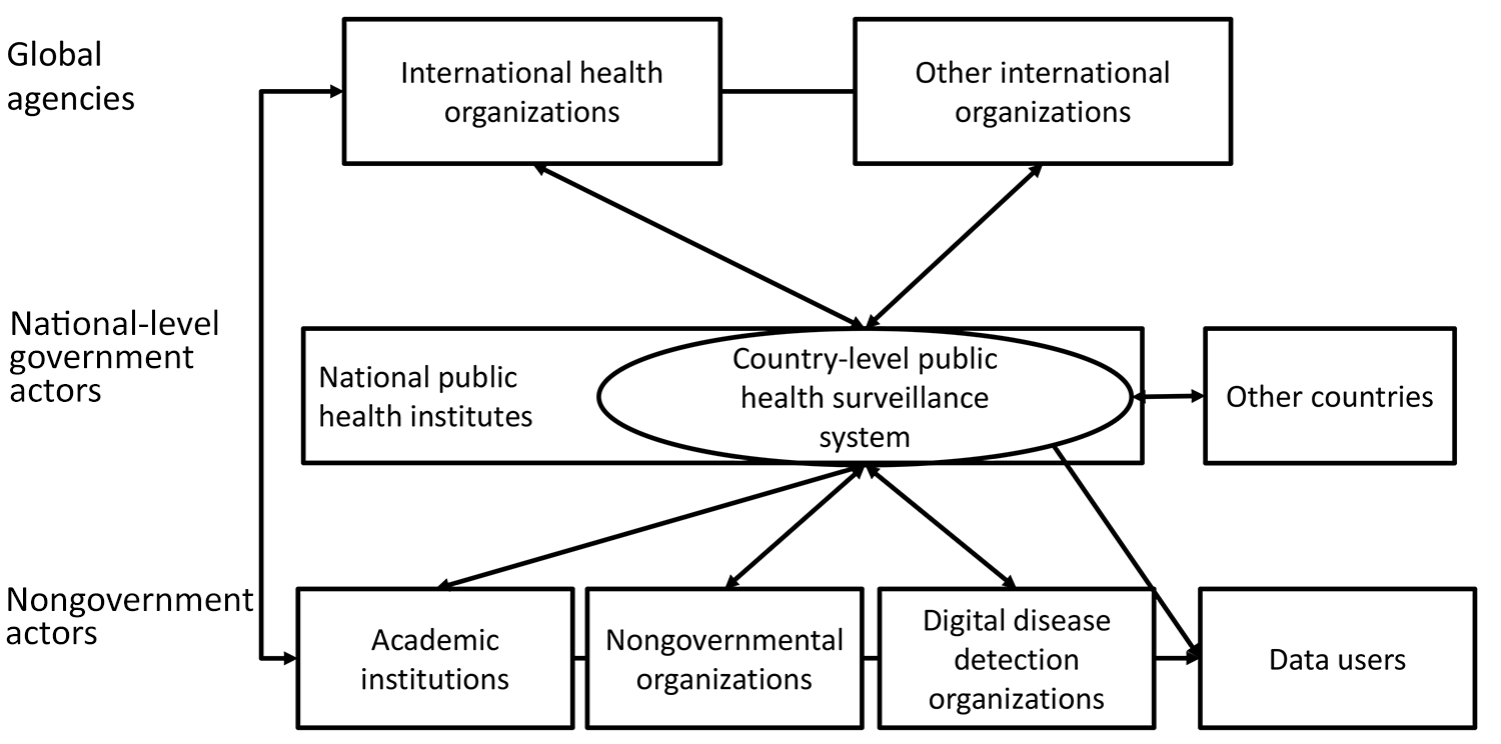
The global public health surveillance landscape, 2018.

Stakeholders can be divided into 3 groups that need to be engaged for optimal data sharing: 1) data providers, who generate public health surveillance data either from the community, the healthcare system, or nonhealth sources; 2) data recipients, who interpret and use data generated by others; and 3) data sharing facilitators, those who make sharing between data providers and recipients possible.

Individual stakeholders can commonly belong to >1 group at a time and can assume a different role in different situations. For example, a country might provide surveillance data to a multilateral agency and receive data from a neighboring country simultaneously; alternatively, a multilateral agency might provide data sharing guidelines to countries, acting as facilitator, while also receiving and analyzing event-based data.

Disease outbreaks caused by Ebola virus (2014–2016) and Zika virus (2016), among others, have increased awareness of the importance of data sharing among global health stakeholders. In addition to several other recent calls to share research data during emergencies (*30*,*31*), in 2016, a group of ≈40 international public health leaders published a statement calling on stakeholders to share “all public health surveillance data, as necessary to improve and protect public health” (*18*; Technical Appendix). This statement further outlined that public health surveillance data sharing should be the norm, rather than the exception, with public health surveillance data made accessible in a timely manner while taking appropriate steps to safeguard the privacy of individuals and other legitimate public interests. Accordingly, the statement asked stakeholders to commit to 1) sharing public health surveillance data by default when a public health need is identified; 2) using public health surveillance data responsibly, with the intention of protecting and improving the health of the population; 3) making the benefits explicit; 4) ensuring that public health surveillance data are shared with as few restrictions as possible and in an ethical way.

## Principles for Sharing Public Health Surveillance Data

Formulating principles to promote and facilitate data sharing in public health is not a new concept. An example is the Pandemic Influenza Preparedness Framework (*32*). This guidance document is restricted to 1 disease and resulted only after extensive negotiations (*32*). In addition to such international efforts, individual funders often have specific data-sharing policies (*33*).

The principles we outline here are the result of a consultation process with ≈100 experts, including those from the fields of public health, law, ethics, politics, and data sharing, including experts from WHO, the World Organisation for Animal Health (OIE), and the Food and Agriculture Organization of the United Nations. The consultation was convened by the Chatham House Centre on Global Health Security (*34*). The principles form the basis of a guide to sharing public health surveillance data and benefits. This guide, available in hard copy and as an online interactive tool (https://datasharing.chathamhouse.org), addresses perceived and real barriers and is intended to facilitate equitable sharing of public health surveillance data and benefits. Equitable in this context is taken to mean that data and benefits are shared among stakeholders according to individual, organizational, and public health needs. The guide enables sharing without the need for prolonged negotiation by creating an environment conducive to sharing data and achieving good practice.

## The Principles

This approach is governed by 7 principles. Each incorporates the ethical concepts most relevant to data sharing: social beneficence, respect, justice, and transparency. Those principles also strive to ensure that sharing data when a need is identified leads to equitable sharing of public health benefits and capacity-building where necessary and appropriate. This component is particularly important when the parties sharing the data have different capacities to benefit because of unequal resources. The principles encourage parties who are better resourced to ensure that others benefit from the process according to need.

### 1. Building Trust

Trust facilitates successful data sharing, which in turn further reinforces trust. Two principal dimensions to trust are brought to bear when public health surveillance data are shared. First, organizations sharing public health surveillance data should do so in a transparent manner and should be able to demonstrate to communities from which the data originate how their data are collected, analyzed, used, and protected. Second, trust-building measures between organizations or individuals sharing data, whether at the personal or organizational level, help create an environment where public health surveillance data can be shared. Transparency with regard to what data are shared with whom, and for what purpose, is a prerequisite. Trust includes ensuring that the shared data are used responsibly and not made available to other parties or publicly without consulting the data providers. When the purpose of sharing data is clear and explicit, and those persons involved in sharing know each other, understand each other’s expectations, and carry out commitments as agreed, a trust relationship can emerge. Established trust increases the likelihood of collaboration and shared benefits and promotes core surveillance capacity through the creation of surveillance networks (*19*). Building trust for routine data sharing can provide strong foundations for emergency surveillance and response. Building trust can be hard, but losing trust is all too easy.

In practice, trust often translates into developing appropriate professional relationships with counterparts in other countries or regions (*35*). Trust-building measures sometimes take the form of face-to-face meetings, regional workshops, desktop exercises, or joint outbreak investigations (*19*). For data recipients, providers must be trustworthy in providing high-quality data. Therefore, improving data quality through capacity-building (for example, by sharing technical expertise) is in their interest.

### 2. Articulating the Value

The benefits of sharing should be explicitly articulated when public health surveillance data are shared. However, loss of rights over the data and the potential for misuse can increase the risk of data providers being reluctant to share because of real or perceived reputational damage and loss of benefits, either in terms of public health or, for example, publication opportunities (*35*). Because of these potential negative outcomes, some public health authorities have used legal or operating standards to restrict data sharing (*36*). When initiating data sharing, the purpose must be explicit, and all stakeholders should be able to understand the value of sharing the data, who will have access to the data, and how the data will be used. Stakeholders must also be assured that they will benefit from the sharing process in an equitable manner in terms of collaboration opportunities and public health benefits. In situations where no direct benefits to the data providers exist, the sharing process should ensure that, at a minimum, those providing data do not suffer adverse public health consequences or lose opportunities for publication, collaboration, or otherwise.

Such assurances maximize the utility of the data while allowing data providers to retain control over the data, thereby encouraging data sharing. Any data use viewed as data harvesting (i.e., when data recipients use data while no benefit is enjoyed by the data provider) is unjust and unfair. Such a practice increases reluctance to share and jeopardizes sharing globally. Conversely, organizations claiming ownership of, and restricting access to, public health surveillance data when such actions would decrease potential health benefits derived from those data is unacceptable.

### 3. Planning for Data Sharing

Public health surveillance data should be collected with potential sharing in mind. Sharing is most successful when expectations of all stakeholders are met and it addresses a need, whether real or perceived (*35*), which should be identified in advance to help ensure timeliness of sharing.

An a posteriori approach to sharing might not maximize benefits, particularly when timeliness is a key element of success, such as in emergencies. Planning for data sharing extends to all steps of the data-management lifecycle (i.e., data collection, processing, analysis, preservation, access, reuse, and disposal) (*37*). This effort requires technical capacity, information technology infrastructure, and a workforce with data-management skills.

Planning also requires a professional ethic for responsibility to protect identifiable data, which are often collected without individual consent. Preserving confidentiality of individual-level data is critical because societies can sometimes respond to persons with infectious diseases in stigmatizing and discriminatory ways.

Data-sharing and data-management standards, in particular with regard to metadata, help maximize quality, utility, and reuse potential. Data recipients benefit from high standards, which ensure that they will be able to reuse data according to their agreed purpose. The time and skills required to collect and manage data in adherence with relevant standards should be taken into account when hiring and training staff. Having a data provider with the human resource and technical capacity to provide the data to required standards is in the data recipient’s interest. As such, data sharing can be an opportunity for IHR 2005–mandated capacity-building.

### 4. Achieving Quality Data

High-quality data enable the generation of high-quality evidence and therefore lead to better public health outcomes. Surveillance data can be evaluated for relevance, accuracy, timeliness, accessibility, interpretability, and coherence, among other characteristics (*38*). Generally, trade-offs exist between these characteristics, and the attributes to prioritize should be considered when sharing the data. Overall, data accessibility and sharing subject the data to feedback and therefore improves quality. Technical and human resource implications of data quality exist; for example, standardization and automation can make sharing less expensive, more effective, and easier (*35*). Standardization also improves the validity and public health benefit of comparative analyses, which are particularly challenging to interpret if data from nonstandardized surveillance systems are aggregated. High-quality data production requires a skilled workforce to develop, manage, and evaluate surveillance systems (*35*). However, when a public health situation warrants the rapid sharing of data, concerns about quality should not be a reason not to share, providing sufficient confidence in the data to inform public health action exists. Quality should be balanced with timeliness.

### 5. Understanding the Legal Context

The legal implications of data sharing and the most suitable type of agreement depend on geographic location, type of institution involved, type of data, level of public health threat, and other contextual factors. Parties should understand the legal implications and tools available. Sharing public health surveillance data across borders has legal implications when the type of data shared is protected by national or international law. This concerns applies mainly to disaggregated data containing confidential or personal information. In current practice, guidance on the legal implications of cross-border public health data sharing is not readily available. Where this guidance does exist, the balance between making data accessible, safeguarding privacy, and protecting intellectual property is not well regulated or standardized, which can result in protective policies (*27*). Governments are often more likely to focus on safeguarding their institutions against liability when creating agreements, whereas nongovernment institutions sometimes focus more on intellectual property concerns.

Data-sharing agreements can help resolve differences or ambiguities in law and are most successful when the context is defined as precisely as possible, supported by local knowledge, and when relevant laws and regulations are taken into account. In some instances, an agreement that is not legally binding may be more suitable than using legal means.

### 6. Creating Data-Sharing Agreements

Formal data-sharing agreements are unnecessary if informal arrangements are sufficient to accomplish the goal of sharing. The rights and interests of stakeholders should be properly taken into account whatever arrangements are made. When more formal agreements are required, they can take different shapes, from short memoranda of understanding to detailed, legally binding data-sharing agreements. Depending on the context, the agreement can take place at the local, national, regional, or global level. Whatever form they take, successful and sustainable data-sharing agreements require consideration of the needs and expectations of all parties. Agreements drafted before the needs and expectations of all parties are understood can lead to inequities in the sharing of benefits (*35*). This imbalance can also result in missed opportunities for knowledge and skills capacity-building. Parties should collaborate and ensure that the terms of reference are acceptable to all, data providers have the opportunity to take part in any data analysis if they wish so, benefits are shared equitably, and potential harms to individuals and communities are minimized. Tools and resources to help parties initiate or revise data sharing agreements are available online (https://datasharing.chathamhouse.org)

### 7. Monitoring and Evaluation

Sharing data only leads to public health benefit if a need is addressed and the data are visible and usable. Therefore, it is important to ensure that the data are shared according to the plan, used for the intended purposes once they have been shared, and achieve the desired effect. If these outcomes are not achieved, the reasons should be analyzed. As new sources of surveillance data emerge and as data are successfully shared, recording and disseminating success stories that demonstrate the added value of data sharing also are important. These stories can help trigger a “norm cascade” that creates a critical mass of stakeholders who adopt data sharing as a normative expectation (*39*). In addition, in situations where sharing did not have the expected result or a lack of data sharing contributed to negative public health outcomes, the process should be documented and analyzed to help understand and make improvements in the future.

## Conclusions

Sharing surveillance data improves public health. We propose an approach to data sharing that creates an environment conducive to sharing, encourages good practice, and ensures that the benefits derived from the sharing process are equitably distributed.

The public health surveillance landscape is complex, with a range of government and nongovernment stakeholders who can provide and receive data as well as facilitate sharing. Optimal sharing requires an understanding of the roles and responsibilities of these stakeholders. The 7 principles for public health surveillance data sharing we propose address good practice for the sharing of public health surveillance data. Those principles serve as the basis for comprehensive guidance with actionable recommendations for all stakeholders. The complete guidance is available online (https://datasharing.chathamhouse.org).

Sharing of public health surveillance data is best done with an agreement that takes into account those principles, which will help to ensure that data are shared optimally and ethically, while fulfilling the expectations of stakeholders and facilitating equitable distribution of benefits. We encourage stakeholders, and in particular multilateral organizations, to consider these principles when strengthening frameworks and capacity for data sharing.

Technical Appendix Statement on public health surveillance and the call to share data.
