# Supplementary material for: Strengthening Global Public Health Surveillance through Data and Benefit Sharing
Source: Emerg Infect Dis. 2018 Jul;24(7):1324–30. doi: 10.3201/eid2407.151830 (PMC6038731; doi:10.3201/eid2407.151830)
Supplement: Technical Appendix — Statement on public health surveillance and the call to share data. [file 15-1830-Techapp-s1.pdf]

Article DOI: https://doi.org/10.3201/eid2407.151830

Strengthening Global Public Health Surveillance through Data and Benefit Sharing

Technical Appendix

Public Health Surveillance: A Call to Share Data

IANPHI US Office

Disease outbreaks such as Ebola, and increasing concerns about the impact of Zika virus, point to the urgent need for sharing public health surveillance data, including that collected by governments, researchers, donors and international organizations. Convened by Chatham House, over 40 international public health leaders, including IANPHI Founding President Dr. Jeff Koplan, Vice President Dr. Mwele Malecela and Executive Board member Dr. Ilesh Jani participated in the discussion and development of the statement.

“Sharing routine public health surveillance data is crucial to understanding what is going on in our countries, regions and the world as a whole,” said Dr. Malecela, who is the director of the National Institute of Medical Research, Tanzania. “We commend Chatham House for its important work in bringing together thought leaders to develop strategies for the future.” Dr. Jani noted that public health institutes such as Mozambique’s National Institute of Health play a major role in analyzing surveillance data and developing policy recommendations. “To do so we need access to all types of data, including that from those working in our countries.” Dr. Koplan noted that the new Child Health and Mortality Prevention Surveillance network (CHAMPS), in which IANPHI is a partner, will result in substantial new data on the causes of childhood death. “This data, in line with our discussions at Chatham House, will be widely available in a way that will help parents, communities, nations and the world,” he said. IANPHI members interested in signing the document may do so by emailing us at communications@ianphi.org.

The beginning of the 21st century has seen several global disease outbreaks including SARS, H1N1, Ebola and Zika. These outbreaks differ in many ways, but they all have one

important characteristic in common: timely sharing of public health surveillance data as the outbreaks unfolded led to more coordinated and effective risk management and improved the public health response. Conversely, as the MERS outbreak has illustrated, a failure to share can have very real health consequences at the individual and population levels.

Sharing Data Saves Lives

The benefits are not restricted to outbreaks. Sharing routine public health surveillance data enables regional collaborations, capacity strengthening, insight into public health system performance and ultimately better control of infectious diseases. For example, timely and accurate sharing of polio surveillance data has clearly contributed to the reduction in circulation of wild polio virus on the way to eradication. In addition, the global sharing of antimicrobial resistance data enables the tracking and mitigation of what is considered one of the biggest health threats of the 21st century.

Data Sharing will be Essential for the Achievement of the Sustainable Development Goals

Successful control of infectious diseases benefits individuals and communities globally and can be considered a global public good. Public health surveillance is a critical tool that helps achieve this. Nevertheless, despite examples of success, sharing public health surveillance data beyond national borders is still not the norm. There are understandable technical, political, ethical, legal and economic reasons why there may be reluctance to share. However, restricting access can harm the health of populations and denies health benefits to individuals entitled to them. Thoughtful deliberate policies that protect legitimate interests without restricting access are urgently needed.

Since 2014, the Centre on Global Health Security at Chatham House has worked to overcome the obstacles by developing guidance on how to create the right environment and achieve good practice for sharing data for public health action. The Strengthening Data Sharing for Public Health project' has been an iterative process involving a wide range of leading experts and targeted thematic and regional roundtables.

Those who have engaged in the Chatham House project recognize that public health surveillance data benefit not only those to whom the data directly relate, but also the wider community. Sharing these data helps to inform local and global decision making to achieve better health outcomes. Noting the recent calls for the sharing of data for research and during

emergencies, we now call for all public health surveillance data to be shared as necessary to improve and protect public health. We also consider it unacceptable for organizations to claim ownership of, and restrict access to, public health surveillance data when that would decrease potential health benefits derived from these data. The norm should be for these data to be accessible in a timely manner for public health action while taking appropriate steps to safeguard the privacy of individuals and other legitimate public interests.

The consequences of making a decision to withhold data can be critical, and those considering such a decision must be ready to justify their actions. In accordance with the recent Statement on Data Sharing in Public Health Emergencies, the sharing of data in advance of publication in peer-reviewed journals should not be penalized.

We call on stakeholders to commit to the following:

- To share public health surveillance data by default where a public health need is identified, in a timeframe necessary for public health decision-making and to the highest standards they can achieve.
- To use public health surveillance data responsibly, with the intention of protecting and improving the health of the population and in accordance with the agreed terms.
- To articulate the value proposition for sharing in an explicit, clear, and accessible way - the benefits should be evident to all.
- To ensure that public health surveillance data are shared with as few restrictions as possible, and with a commitment to principles of social beneficence, respect, justice and transparency.

We believe strongly that real and perceived obstacles to sharing can be overcome by working together, and that establishing sharing as the new norm will bring great benefit in improving and protecting public health.

Signatories to the Statement

Dr. Clement Adebamowo, Director of Strategic Information, Research and Training, Institute of Human Virology, Nigeria

Dr. Obijiofor Aginam, Senior Research Fellow, International Institute for Global Health, United Nations University

Dr. Ray Arthur, Director, Global Disease Detection Operations Cente, U.S. Centers for Disease Control and Prevention (CDC)[1]

Dr. Daniel Barth-Jones, Assistant Professor of Clinical Epidemiology, Mailman School of Public Health, Columbia University

Mr. Eric Beda, Regional ICT Specialist, Southern African Centre for Infectious Disease Surveillance (SACIDS)

Dr. Louise Bezuidenhout, Research Fellow, College of Social Sciences and International Studies, University of Exeter

Prof. Martin Bobrow, Emeritus Professor of Medical Genetics, University of Cambridge and Global Alliance for Genomics and Health

Mr. Matthew Brack, Project Manager, Centre on Global Health Security, Chatham House

Dr. Robert Breiman, Director, Emory Global Health Institute, Emory University

Prof. John Brownstein, Co-founder, HealthMap

Mr. David Carr, Policy Advisor, Wellcome Trust

Ms. Lisa Carlson, Associate Director of Management and Operations, Emory Global Health Institute, Emory University

Prof. Richard Coker, Professor of Public Health and Head, Communicable Diseases Policy Research Group, London School of Hygiene & Tropical Medicine

Ms. Jennifer Cole, Postgraduate Research Scientist, Department of Computer Science, Royal Holloway, University of London

Prof. Yali Cong, Professor of Medical Ethics, Institute of Medical Humanities, Peking University Health Science Centre

Dr. Sara Davies, ARC Post-doctoral Fellow, Centre for Governance and Public Policy, Griffith University

Dr. Yves-Alexandre de Montjoye, Post-doctoral Researcher, The Institute for Quantitative Social Science, Harvard University

Dr. Michael Edelstein, Consulting Research Fellow, Centre on Global Health Security, Chatham House

Dr. Claudia Emerson, Director Program on Ethics & Policy for Innovation, McMaster University

Dr. Debra Hanna, Executive Director, Critical Path to TB Drug Regimens, Critical Path Institute

Prof. David Harper CBE, Senior Consulting Fellow, Centre on Global Health Security, Chatham House

Dr. Habib Hasan, Associate Professor, Indian Institute of Public Health-Delhi, Public Health Foundation of India

Ms. Asha Herten-Crabb, Project Coordinator, Centre on Global Health Security, Chatham House

Prof. David Heymann CBE, Head and Senior Fellow, Centre on Global Health Security, Chatham House

Prof. Unni Karunakara, Senior Fellow Jackson Institute for Global Affairs, Yale University; Former International President, Médecins Sans Frontières[2]

Dr. Stephen Keenan, Global Alliance Working Group Manager, European Bioinformatics Institute and Global Alliance for Genomics and Health

Prof. Ann Marie Kimball, Senior Consulting Fellow, Centre on Global Health Security, Chatham House

Dr. Francis Kombe, Training Coordinator (Bioethics and Communication), Kenya Medical Research Institute (KEMRI)-Wellcome Trust Research Programme

Dr. Jeffrey Koplan, Vice President for Global Health, Emory University; Co-founder, International Association of National Public Health Institutes (IANPHI)

Dr. Shengjie Lai, Division of Infectious Diseases, Chinese Center for Disease Control and Prevention

Ms. Katherine Littler, Senior Policy Advisor, Wellcome Trust

Dr. Sarah Macfarlane, Professor, School of Medicine, University of California San Francisco

Dr. Larry Madoff, Editor, ProMED-mail

Dr. Moe Ko Oo, Coordinator, Mekong Basin Disease Surveillance Foundation (MBDS)

Ms. Emma Orefuwa, Programme Manager, Connecting Organizations for Regional Disease Surveillance (CORDS)

Dr. Marisella Ouma, Former Executive Director, Kenya Copyright Board

Dr. Charles Rotimi, Director, Center for Research on Genomics and Global Health, U.S. National Institutes of Health (NIH)

Prof. Osman Sankoh, Executive Director, INDEPTH Network

Dr. Samuel Scarpino, Assistant Professor, Vermont Complex Systems Center, University of Vermont

Dr. Murray Stewart, Chief Medical Officer, GlaxoSmithKline

Dr. Laura Streichert, Executive Director, International Society for Disease Surveillance

Dr. Kumnuan Ungchusak, Senior Expert, Department of Disease Control, Thailand Ministry of Public Health

Dr. Effy Vayena, Division Head, Epidemiology, Biostatistics and Prevention Institute, University of Zurich

Mr. Sean Ward, Founder & Chief Technology Officer, Synthace

Dr. Julia Wilson, Associate Director, Wellcome Trust Sanger Institute and Global Alliance for Genomics and Health

Prof. Jimmy Whitworth, Professor of International Public Health, Department of Infectious Disease Epidemiology, London School of Hygiene and Tropical Medicine

Ms. Lynn Woolfrey, Manager, DataFirst, University of Cape Town

Prof. Madhav Marathe, Professor, Biocomplexity Institute of Virginia Tech

Prof. Terry Dwyer, Executive Director, The George Institute for Global Health, University of Oxford

Dr. Kendall Krause, Program Officer, Surveillance and Epidemiology, Bill & Melinda Gates Foundation

Dr. Scott Dowell, Deputy Director, Surveillance and Epidemiology, Bill & Melinda Gates Foundation

Mr. Dan Kim, Technology Advisor, Bill & Melinda Gates Foundation

Mr. Richard Wilder, Associate General Counsel, Bill & Melinda Gates Foundation

Prof. Abdulsalami Nasidi, Director, The Nigeria Centre for Disease Control

Dr. Khadija Innocensia Yahya-Malima, Chief Research Officer (Health), Tanzania Commission for Science Technology

Dr. Samba Ceesay, Director of Health Services, The Gambia Ministry of Health

Dr. Bakary Sylla, Project Director, West African Network for Infectious Disease Surveillance (WANIDS), Connecting Organizations for Regional Disease Surveillance (CORDS)

Dr. Zacharie Tsala Dimbuene, Demographer-Statistician, African Population and Health Research Center

Prof. Kwadwo Koram, Director, Noguchi Memorial Institute for Medical Research, University of Ghana

Dr. Michael Conway, Assistant Professor, Department of Biomedical Informatics, University of Utah School of Medicine

Dr. Sok Touch, Director, Communicable Disease Control Department, Cambodia Ministry of Health

Mr. Kim Sampson, Chief Executive Officer, Immunisation Coalition

Dr. Nguyen Dang Vung, Vice Director, Institute for Preventative Medicine and Public Health, Hanoi Medical University

Prof. Abul Khair Mohammad Shamsuzzaman, Director, Disease Control, Bangladesh Directorate General of Health Services

Dr. Paba Palihawadana, Director, Central Epidemiologic Unit, Sri Lanka Ministry of Health

Prof. Woo Joo Kim, Professor, Division of Infectious Diseases, Korea University Guro Hospital

Dr. Foday Dafae, Director, Disease Prevention and Control, Sierra Leone Ministry of Health

[1]The findings and conclusions in this report are those of the authors and do not necessarily represent the official position of the Center for Disease Control and Prevention.

[2]The findings and conclusions in this report are those of the authors and do not necessarily represent the official position of Médecins Sans Frontières.
